# Development of molecular and pharmacological switches for chimeric antigen receptor T cells

**DOI:** 10.1186/s40164-019-0151-z

**Published:** 2019-11-05

**Authors:** Bill X. Wu, No-Joon Song, Brian P. Riesenberg, Zihai Li

**Affiliations:** Division of Medical Oncology, Pelotonia Institute for Immuno-Oncology, The Ohio State University Comprehensive Cancer Center, Columbus, OH 43210 USA

## Abstract

The use of chimeric antigen receptor (CAR) T cell technology as a therapeutic strategy for the treatment blood-born human cancers has delivered outstanding clinical efficacy. However, this treatment modality can also be associated with serious adverse events in the form of cytokine release syndrome. While several avenues are being pursued to limit the off-target effects, it is critically important that any intervention strategy has minimal consequences on long term efficacy. A recent study published in Science Translational Medicine by Dr. Hudecek’s group proved that dasatinib, a tyrosine kinase inhibitor, can serve as an on/off switch for CD19-CAR-T cells in preclinical models by limiting toxicities while maintaining therapeutic efficacy. In this editorial, we discuss the recent strategies for generating safer CAR-T cells, and also important questions surrounding the use of dasatinib for emergency intervention of CAR-T cell mediated cytokine release syndrome.

The validation of chimeric antigen receptor (CAR) T cell technology as a viable, therapeutic strategy for the treatment of blood-born human cancers has resulted in a renewed focus on ways to optimize and control this treatment modality [1, 2]. CAR constructs are generated by linking an extracellular antibody-antigen recognition fragment with a costimulatory and intracellular activation domain from the T cell receptor complex, allowing for T cell recognition and clearance of tumors. The FDA has recently approved two CD19-directed CAR-T cell programs, Tisagenlecleucel (Kymriah) and Axicabtagene ciloleucel (Yescarta), for the treatment of various B cell malignancies including diffuse large B cell lymphoma (DLBCL) and acute lymphoblastic leukemia (ALL). As is the case with other cancer treatments, CD19-CAR-T cell therapy can also demonstrate serious, adverse side effects, the most common of which being cytokine release syndrome (CRS). CRS is characterized by massive and rapid releases of cytokines into the bloodstream, resulting in morbidity and even mortality in some patients. Different from other traditional cancer drugs, however, CAR-T cells are considered “living drugs” which may reside in patients for years, thereby increasing the probability for undesirable effects. Thus, development of precise techniques to manage the balance of activity and toxicity of CAR-T cells is of extreme importance.

Multiple strategies have been investigated experimentally, largely focusing on alterations to the CAR construct design, in order to generate safer CAR-T cell therapy (Table 1). Briefly, these strategies include introduction of suicide genes, dual targeted activation, inhibitory modules, and modification of structure to separate cytokine release from cytolytic signals. Incorporation of suicide genes, such as inducible caspase 9 (iCasp9) [3] and truncated epidermal growth factor receptor (EGFRt) [4], enables successful elimination of CAR-T cells in the event of abnormal activation by auxiliary treatment of patients with AP1903 or cetuximab, to activate iCasp9 or to target EGFRt respectively. Dual targeted activation strategy requires two tumor specific antigens recognized by CAR-T cells to be activated. This two-antigen recognition system helps CAR-T cells to avoid undesired activation by normal tissues while simultaneously aiding in anti-tumor activity and specificity of CAR-T cells [5]. Similar to dual targeted activation, inhibitory CAR molecules contain an antigen recognition domain specific to the antigens expressed by normal tissues. Subsequent interaction between antigen and receptor in this setting arrests CAR-T cell activity [6]. Modifying the CAR construct has also been shown to provide safer treatment with CAR-T cells without diminishing clinical efficacy. Using a tertiary-structure-prediction program, Ying and colleagues were able to limit the toxicities associated with CAR-T cell therapy in a phase I clinical trial by altering the length of the transmembrane domain [7]. While these preliminary findings are exciting, uncovering a pharmacological intervention strategy allows for in vivo control of adverse toxicities while maintaining the anti-tumor response is an important endeavor.

**Table 1:** Strategies for safer CAR-T therapy

Strategy	Mechanism
Suicide gene	Incorporation of suicide genes (iCasp9, EGFRt) [3, 4]
Combinational targeted activation	Two antigen recognitions are required to fully activate CAR-T cells [5]
Inhibitory CAR-T cells	Recognition of normal cell induces inhibition of CAR-T cell activity [6]
CAR modification	Modification of CAR molecules to identify constructs with less cytokine production activity but with preserved anti-tumor function [7]
On/off switch for CAR-T cell	Dasatinib serves as an on/off switch for CAR-T cells [8]

In a recent study published in Science Translational Medicine, Mestermann et al. showed that dasatinib, a tyrosine-kinase inhibitor, mediated functional suppression of both CD4^+^ and CD8^+^ CD19-CAR-T cells in a dose-dependent manner [8] (Fig. 1). In vitro analysis with dasatinib demonstrated superior control over CAR-T cell function when compared to that of dexamethasone, an anti-inflammatory steroid used clinically to combat CAR-T cell toxicity [9]. From a mechanistic standpoint, dasatinib treatment abrogated phosphorylation of multiple key components in the CAR signaling domain, including lymphocyte specific protein tyrosine kinase (LCK), CD3ζ, and ZAP70. Importantly, the inhibitory effects were reversible as depletion of dasatinib immediately recovered CAR-T cell function and proliferation, both in vitro and in vivo, without compromising the long term efficacy of therapy. Finally, the authors validated dasatinib’s potential as an emergency intervention strategy by using an acute CRS onset model [10]. In this setting, mice developed lethal CRS 2–3 days after CAR-T cell transfer. By initiating dasatinib 3 h after CAR-T transfer and continuing treatment for the next 30 h, the authors were able to significantly enhance the immediate survival rate of mice. Taken together, the work presented by Mestermann et al. makes a convincing case for the potential use of dasatinib as an emergency drug to counter CAR-T mediated CRS in patients.

**Fig. 1:**
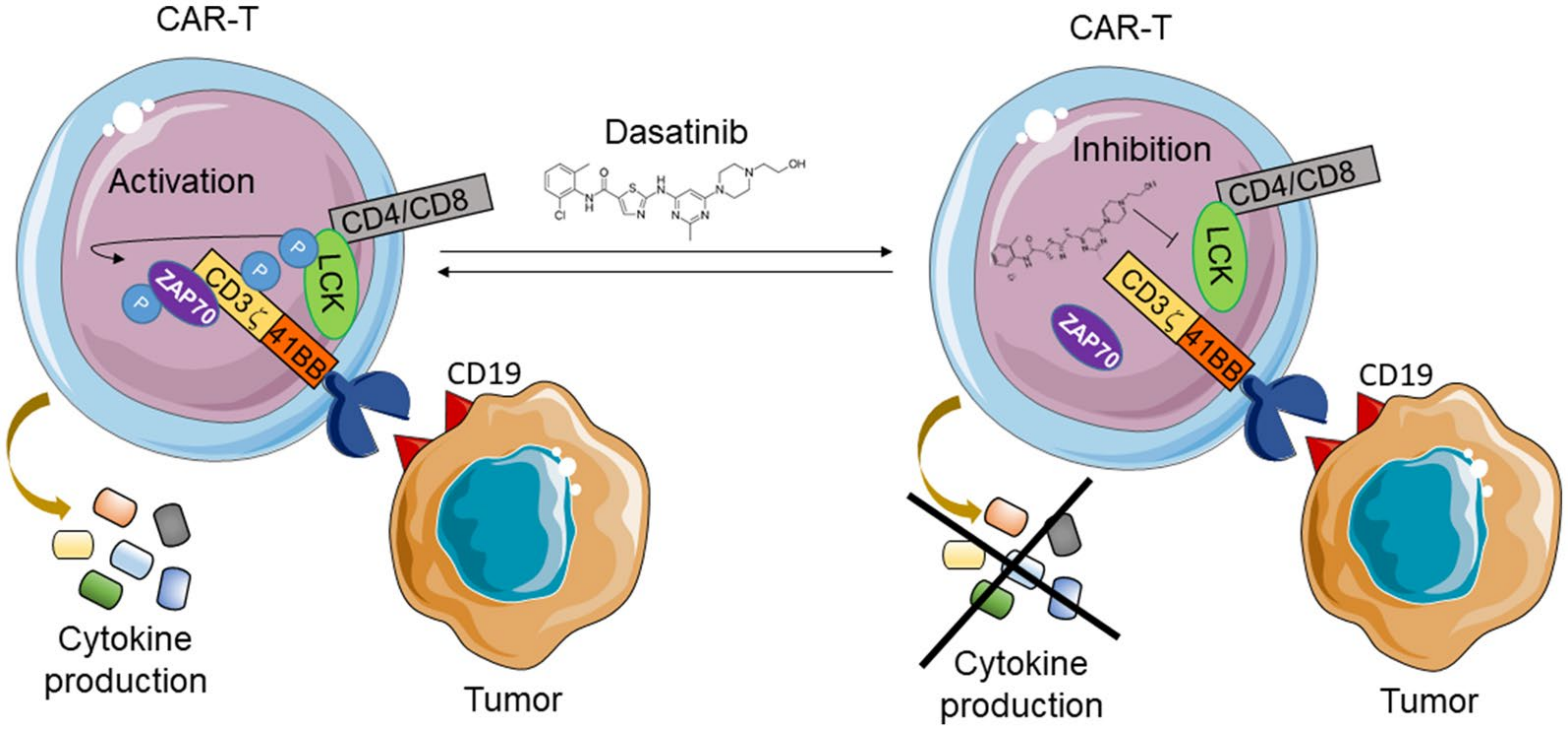
Dasatinib, a tyrosine kinase inhibitor against BCR-ABL, can inhibit the phosphorylation and activation of components in the T cell receptor signaling pathways. It was shown to be effective in reversing CD19-CAR-T cell mediated cytokine release syndrome in a recently published preclinical model [8]

As mentioned above, dasatinib is a tyrosine-kinase inhibitor which has been highly effective in treating patients with imatinib-resistant chronic myeloid leukemia (CML) and Philadelphia chromosome-positive ALL. However, as a nonspecific inhibitor, dasatinib has also been shown to interfere with other kinase family’s function such as Src, Btk, and cKit to name a few [11]. Given the exciting work from Mestermann et al., several questions should be investigated prior to adopting the clinical use of dasatinib for controlling CAR-T cell toxicity. First, understanding how dasatinib influences CAR-T cell function in an immune competent setting should be examined. Current standard of care for CAR-T therapy requires a lymphodepletion regimen allowing for enhanced donor T cell engraftment. Considering the complexity of immune reconstitution in this setting, it would be worthwhile to investigate the effects of dasatinib on this process. Secondly, how dasatinib treatment affects the endogenous tumor-specific T cell response should be addressed. The authors showed dasatinib had similar inhibitory effects when stimulating with an endogenous TCR-peptide combination in addition to stimulation with CD19, validating the broad inhibitory properties of the treatment. However, it is unclear if CAR-T cells are more sensitive to dasatinib compared to endogenous T cells due to increased antigen affinity as well as unique configuration of signaling cassette. Finally, it will be interesting to determine if other tyrosine kinase inhibitors have superior effects in inhibiting CAR-T cell mediated CRS by acting as a functional on/off switch.

The current paper from Dr. Hudecek’s group proved that the tyrosine kinase inhibitor dasatinib can serve as an on/off switch for CD19-CAR-T cells in the preclinical models [8]. Clinical studies with dasatinib for the treatment of CRS induced by CAR-T therapy should be approached with caution until more can be elucidated surrounding the potential for off target effects. Nonetheless, the work presented by Mestermann and colleagues offers an enticing approach for reducing toxicity while maintaining efficacy of CAR-T cell therapy.

## Data Availability

Not applicable.
